# Electrochemical protein biosensors for disease marker detection: progress and opportunities

**DOI:** 10.1038/s41378-024-00700-w

**Published:** 2024-05-22

**Authors:** Lanpeng Guo, Yunong Zhao, Qing Huang, Jing Huang, Yanbing Tao, Jianjun Chen, Hua-Yao Li, Huan Liu

**Affiliations:** 1 School of Integrated Circuits, Wuhan National Laboratory for Optoelectronics, Optics Valley Laboratory, Huazhong University of Science and Technology, Wuhan, 430074 China; 2 Key Laboratory of Intelligent Computing and Signal Processing of Ministry of Education, School of Integrated Circuits, Anhui University, Hefei, 230601 China; 3 School of Optoelectronic Materials and Technology, Jianghan University, Wuhan, 430056 China; 4 Department of Otorhinolaryngology, Union Hospital, Tongji Medical College, Huazhong University of Science and Technology, 1277 Jiefang Avenue, Wuhan, 430022 China; 5 Wenzhou Institute of Advanced Manufacturing Technology, Huazhong University of Science and Technology, Wenzhou, 325000 China

**Keywords:** Biosensors, Electronic properties and materials

## Abstract

The development of artificial intelligence-enabled medical health care has created both opportunities and challenges for next-generation biosensor technology. Proteins are extensively used as biological macromolecular markers in disease diagnosis and the analysis of therapeutic effects. Electrochemical protein biosensors have achieved desirable specificity by using the specific antibody–antigen binding principle in immunology. However, the active centers of protein biomarkers are surrounded by a peptide matrix, which hinders charge transfer and results in insufficient sensor sensitivity. Therefore, electrode-modified materials and transducer devices have been designed to increase the sensitivity and improve the practical application prospects of electrochemical protein sensors. In this review, we summarize recent reports of electrochemical biosensors for protein biomarker detection. We highlight the latest research on electrochemical protein biosensors for the detection of cancer, viral infectious diseases, inflammation, and other diseases. The corresponding sensitive materials, transducer structures, and detection principles associated with such biosensors are also addressed generally. Finally, we present an outlook on the use of electrochemical protein biosensors for disease marker detection for the next few years.

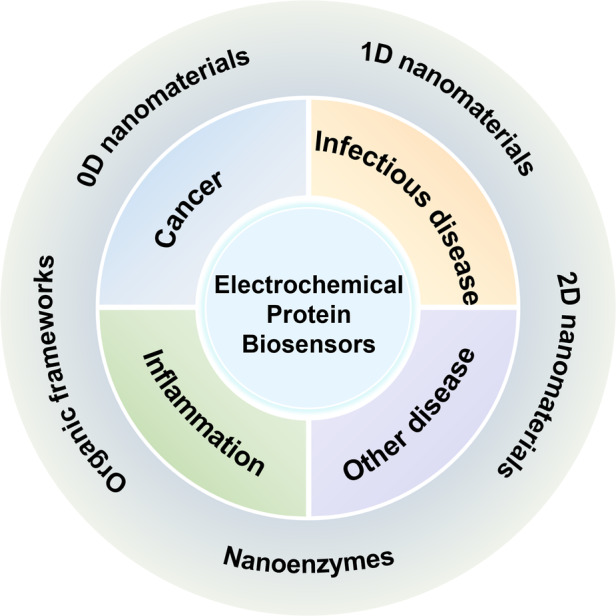

## Introduction

In recent years, with the deep integration of artificial intelligence and smart health care, biosensor technology has undergone unprecedented development. New methods for the rapid detection and online monitoring of human disease markers based on smart biosensors can provide strong support for human life and health^1^. Biosensors are instruments or devices that are sensitive to biological substances and can convert the concentration of these substances into an electrical signal for detection. Biosensors have receptor and transducer functions and consist primarily of a molecular recognition element and a conversion element^2,3^. Compared to traditional commercial immunoassays such as enzyme-linked immunosorbent assay (ELISA)^4^, electrochemical biosensors offer advantages such as high sensitivity, rapid detection speed, low power consumption, and miniaturization^5–7^. Therefore, electrochemical biosensors are a current focus of research in the field of biological detection and analysis.

Biomarkers are distinctive biological or biologically derived indicators that reflect physiological or pathological processes, events, or conditions (aging, disease, etc.) in an organism^8^. Disease markers play a crucial role in early diagnosis, the monitoring of treatment efficacy, and prognosis^9^. Early signs of disease can be identified by detecting specific biomarkers, which can lead to timely treatment. Proteins, such as antigens and antibodies, are widely used as disease biomarkers for diagnostic analyses and the evaluation of therapeutic effects^10,11^. Proteins are a type of biological macromolecule found in all living cells, serving as the fundamental structural and functional components of living organisms. Proteins participate in almost all life processes, and in the form of antibodies, they can defend organisms against disease antigens. Based on the principle of specific antigen–antibody binding in immunology, electrochemical protein biosensors are expected to enable the specific detection and quantitative measurement of protein biomarkers^12,13^. This kind of biosensor can provide a direct or indirect basis for early screening for and detection of diseases, home health care, clinical diagnosis and treatment, disease staging, and prognosis assessment. For example, brain natriuretic peptide (BNP) is a clinical biomarker for heart failure (HF)^14^. The detection of BNP using electrochemical protein biosensors is highly important for the early diagnosis of HF, reducing the risk of recurrence, and alleviating the pain and economic burden of patients. However, electrochemical sensors for protein macromolecular biomarker detection typically present the following challenges: (i) Proteins usually consist of one or more polypeptide chains with a convoluted and complex molecular structure. The structural characteristics of proteins influence long-range electron transfer^15^, which in turn affects charge transfer at the sensor electrode interface. (ii) Due to the low concentration of protein macromolecules, the charge transfer activity during electrochemical detection is limited. As a result, the specific signal is subject to interference by the background signal, which affects the sensitivity and specificity of detection^10^. To address the above issues, researchers have explored methods to improve the long-range electron transfer behavior of proteins and to optimize the charge transfer pathway between proteins and sensor electrodes^12^. Many studies have focused on the chemical modification of electrodes by sensitive materials^16,17^ as well as the structural design of transducer devices^18^ to facilitate the development of electrochemical protein biosensors.

This paper reviews recently reported electrochemical protein biosensors for the diagnosis of cancer, viral infectious diseases, inflammation, and other diseases. The active center of protein biomarkers is surrounded and blocked by the peptide matrix, making charge transfer difficult and resulting in insufficient sensitivity. To address the above problems, researchers are developing electrode modification materials and designing transducer structures to increase the sensitivity, selectivity, and stability of electrochemical protein sensors. The advancement of functional materials and device structures is expected to improve the prospects for practical applications of sensors. In addition, this paper provides an outlook on the trends in the development of electrochemical protein sensors for disease biomarker detection.

## Electrochemical protein biosensors

### Basic principles of electrochemical protein biosensors

**Fig. 1:**
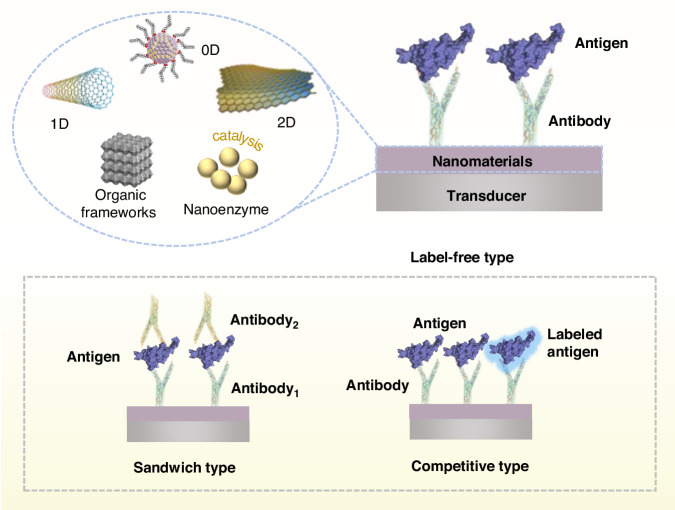
Schematic diagram of the basic principles of nanomaterial-modified electrochemical protein biosensors

Electrochemical protein biosensors are biochemical sensors and constitute an important branch of electrochemical sensing. Similar to traditional biosensors in terms of basic components, electrochemical protein biosensors consist mainly of receptor and transducer functional units^6,19^. The receptor unit performs molecular recognition, and the recognition of biomolecules on its surface causes changes in the physical or chemical properties of the sensitive layer. The receptor units of electrochemical protein biosensors are antigens or antibodies, including both natural antibodies and “plastic antibodies” (such as aptamers and molecularly imprinted polymers^20^). Electrochemical biosensors based on aptamers and molecularly imprinted polymers for detecting protein biomarkers have been described in detail in the relevant literature^2,9,21,22^. This paper focuses on natural antibodies and antigen-based electrochemical protein biosensors for protein disease biomarker detection. The transducer performs biological signal transduction, converting the recognition signals of the receptors into electrical signals (potential, current, impedance, etc.) related to the concentration of the target molecules. These electrical signals enable highly sensitive and specific analysis of the target biomolecules in the samples. The working principle of electrochemical protein sensors (Fig. 1) is as follows^4,23^: taking an antibody as a biological receptor as an example, a protein antigen is the target analyte, and when the antigen to be detected comes into contact with the sensor electrode interface, the specific binding of the antigen and antibody produces an immune complex. Thus, the immune response process is converted into a detectable electrical signal by the transducer, ultimately enabling qualitative and quantitative protein detection^8^.

The recognition of target biomolecules by receptors in electrochemical protein biosensors is based on the mechanism of antigen–antibody binding according to immunological principles^24^. The immunological reaction characteristics of strong specificity and binding between antigen and antibody provide the high specificity and sensitivity of electrochemical protein biosensors. The transducers in electrochemical biosensors are usually electrochemical three-electrode systems and field effect transistors (FETs)^19^. The electrochemical three-electrode system consists of a working electrode (WE), a counter electrode (CE), and a reference electrode (RE). An FET is a three-terminal electronic device that includes a gate, a source, and a drain. By applying a potential to the gate, an electric field action is created in the channel, thereby controlling the current intensity between the source and drain.

### Types of electrochemical protein biosensors and their analysis methods

Electrochemical protein biosensors can be classified into label-free and labeled biosensors^4^. The label-free type detects analyte proteins through a one-step antibody–antigen immunoreaction^4^. The immunoreaction hinders the charge transfer rate at the electrode interface, resulting in a reduced current and increased impedance. Labeled electrochemical protein biosensors can be further divided into sandwich and competitive biosensors^4^. The sandwich-type electrochemical protein biosensor utilizes a labeled detection antibody (secondary antibody, Ab_2_) and an electrode surface-immobilized capture antibody (primary antibody, Ab_1_) to capture the protein antigen, forming a “sandwich”-like structure^25^ that increases the selectivity and sensitivity of the sensor. In competitive electrochemical protein biosensors, sample antigens are mixed with labeled antigens to compete for limited antibody binding sites^25^. The signal of the immune reaction between antigen and antibody is converted into a detectable electrical signal by the labeled substance. The signal intensity generated by the labeled antigen is inversely related to the amount of sample antigen. The main analytical methods based on electrochemical principles include cyclic voltammetry (CV), differential pulse voltammetry (DPV), linear sweep voltammetry (LSV), and electrochemical impedance spectroscopy (EIS)^25,26^. The determination and analysis of target substances are achieved by measuring changes in conductivity, resistance, or capacitance on the electrode surface of the sensor.

### Electrode-modified nanomaterials for electrochemical protein biosensors

In research on protein biomolecule detection, proteins are easily adsorbed on unmodified bare electrode surfaces, which can result in conformational changes and loss of activity^27^. This behavior affects the subsequent specific reaction process and thus the signal transduction of the sensor. Therefore, it is essential to design and construct electrode interfaces with molecular recognition functions for electrochemical biosensors. The properties of biosensors can be improved by modifying the working electrode surface using various functional nanomaterials. Protein biomolecules can be anchored to the surface of functional nanomaterials by labeling methods such as adsorption (direct method)^28^, covalent binding^17^, cross-linking^29^, and entrapment methods^30^. The labeling process should ensure the activity of the biomolecules as well as their stability and durability after labeling. Chemically modified electrodes with specified functions are expected to increase the enrichment of target substances, accelerate the charge transfer reaction, and increase the stability of electrochemical biosensors. The rapid growth of nanotechnology has led to the emergence of nanomaterials with special properties. Nanomaterials usually possess large specific surface areas, quantum size effects, and surface effects^31^, exhibiting unique electrical, optical, mechanical, and catalytic performance. Nanomaterials have several distinct advantages in biosensor research: (i) Nanomaterials act as charge transfer media between electrodes and biomolecules. They can increase the charge transfer rate^23^, especially by increasing the long-range electron transfer of protein biomolecules. (ii) The high specific surface area of nanomaterials is favorable for the modification and immobilization of protein molecules^23^. Moreover, surface chemical modifications can provide a “grip” for anchoring biomolecules and contribute to the binding stability between protein molecules and sensitive films. (iii) Certain catalytic nanomaterials, such as noble metal nanoparticles and nanoenzymes^32,33^, are useful for molecular recognition and signal amplification.

#### Zero-dimensional (0D) nanomaterials

**Fig. 2:**
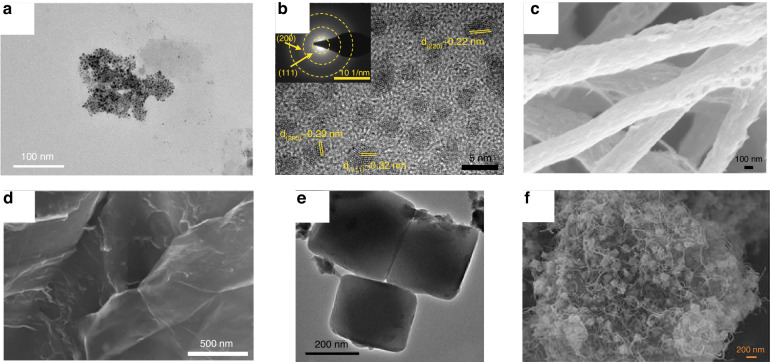
Sensitive nanomaterials for electrochemical protein biosensors. Transmission electron microscopy (TEM) images of **a** glutathione-protected Au NPs^33^ and **b** PbS QDs^12^. Scanning electron microscopy (SEM) images of **c** MWCNT-ZnO nanofibers^40^, **d** aminated graphene oxide^33^, and **e** MOF-808 and **f** MOF-808/CNT composites^49^

0D sensitive materials include metal nanoparticles (NPs), metal oxide nanoparticles, and quantum dots (QDs). Metal NPs such as Au NPs (Fig. 2a), Ag NPs, Pt NPs, and Cu NPs^31,34^ have unique electrical, optical, and catalytic properties. Metal NPs have excellent stability, and suitable ligands can confer good biocompatibility. Li et al.^35^ prepared an electrochemical immunosensor using porous graphene oxide functionalized with Au NPs (p-GO@Au) as the substrate material. Molybdenum disulfide-functionalized multiwalled carbon nanotubes (MoS_2_@MWCNTs) modified with Au@Pd NPs were chosen as the signal-amplifying molecules of the biosensor. The resulting sensor achieved ultrahigh sensitivity for the quantitative measurement of the hepatitis B e antigen and maintained satisfactory detection performance in real samples. Luo et al.^36^ prepared an immunosensor for alpha-fetoprotein (AFP) detection by depositing Cu-Ag nanoparticles onto polydopamine (PDA)-modified cellulose nanofibers. The Cu-Ag nanoparticles effectively increased the electrical signal of the immunocomplex between AFP and the antibody by the electrocatalytic reduction of H_2_O_2_. The limit of detection (LOD) of this biosensor for AFP was 4.27 pg mL^−1^, indicating high specificity. QDs are “quasi-0D” inorganic semiconductor nanoparticles, also known as semiconductor nanocrystals (such as CdS QDs, PbS QDs (Fig. 2b), CdSe QDs, and ZnS QDs), that usually consist of elements of groups II-VI or III-V and typically have diameters of less than 10 nm^37^. QDs have a high specific surface area, and the abundant dangling bonds on their surface can provide a large number of active sites, which is expected to achieve efficient enrichment and labeling of biomolecules on the surface of sensor electrodes^12^. Ortega et al.^38^ constructed an electrochemical immunosensor for IgM antibody detection using PbS QD/polydopamine particle composites as electroactive markers. The sensor had a linear response range of 0–0.5 mg mL^−1^ and an LOD of 0.013 mg mL^−1^ for the measurement of IgM antibodies.

#### One-dimensional (1D) nanomaterials

When the scale of a material in the two-dimensional direction is reduced to the nm level, its morphology is a “fiber”-like structure with unique physicochemical properties different from those of bulk materials. These materials, which are limited to the nm level in the two-dimensional direction, are called 1D nanomaterials. 1D nanomaterials include nanotubes, nanowires, nanorods, nanofibers (Fig. 2c), and others^39,40^. According to the analysis of the conductive properties, electrons move along the axial direction in 1D nanostructures. Moreover, 1D nanomaterials have improved transport properties^41^, which can help to increase the charge collection efficiency and decrease losses during the transport process. In addition, 1D nanomaterials can act as “electronic wires” between electrodes and biomolecules. A small amount of biomolecules can change the electrical conductivity of 1D nanomaterials and increase their sensitivity. Li et al.^42^ used a dual-enhancement strategy combining polydopamine-modified N-doped multiwalled carbon nanotubes (PDA-N-MWCNTs) and graphene/mesoporous Au@Pt nanodendrite composites to achieve ultrasensitive detection of AFP. The PDA-N-MWCNT composites not only achieved good protein labeling but also increased the charge transfer rate at the interface, which greatly increased the sensitivity of the electrochemical immunosensor. Alves et al.^28^ immobilized SARS-CoV-2 recombinant trimeric spike proteins on ZnO nanorods by physical adsorption for the detection of SARS-CoV-2 antibodies. The ZnO nanorods provided an environment that maintained protein activity while facilitating charge transfer. The prepared immunosensor had high sensitivity and excellent specificity.

#### Two-dimensional (2D) nanomaterials

2D materials with excellent surface activity, remarkable electrical properties, good biocompatibility, and high specific surface area have become popular materials in the field of biosensors. Typical 2D materials include graphene (Fig. 2d) and transition metal dichalcogenides (TMDCs)^33,43^. Graphene is a 2D material with sp² hybridized carbon atoms closely arranged to form a honeycomb lattice structure. The unique 2D structure of graphene gives it a large specific surface area and excellent electrical conductivity^44^, and theoretically, all the carbon atoms on the surface can be in contact with the target molecule. Functionalized graphene containing specified functional groups on its surface can be prepared by physicochemical modification to increase the surface activity and biocompatibility^45^. Kim et al.^18^ prepared a graphene FET-based biosensor for the detection of the SARS-CoV-2 spike protein with an LOD of 1 fg mL^−1^. The graphene-based FET biosensor provided highly sensitive and low-noise detection of SARS-CoV-2 antigenic proteins. MoS_2_ is a typical TMDC and consists of 2D stacked layers. The neighboring layers of MoS_2_ are bound together by relatively weak van der Waals forces. Sarkar et al.^43^ designed a MoS_2_-based FET biosensor. The two-dimensional layered structure endows the device with high sensitivity, reaching an LOD of 100 fM for streptavidin. Yáñez-Sedeño et al.^29^ modified reduced graphene oxide (rGO)/MoS_2_/Ag NP nanocomposites and immobilized specific capture antibodies on the electrode surface for the detection of glial fibrillary acidic protein (GFAP). Due to the high conductivity of graphene, the pseudoperoxidase activity of MoS_2_, and the catalytic activity of Ag, the sensor achieved highly sensitive detection of GFAP with an LOD of 0.16 ng mL^−1^. In addition to graphene and MoS_2_, MXene is a 2D material belonging to the family of transition metal carbides, nitrides, or carbon nitrides. MXene has broad application prospects in electrochemical biosensors due to its large specific surface area, excellent electrical conductivity, and good tensile properties^46^. Ahmed et al.^47^ proposed a strategy for the construction of novel electrochemical biosensors using dialdehyde cellulose nanocrystal (DCNC)-Ag NP-functionalized MXene nanocomposites. The resulting DCNC-Ag-MXene nanocomposites increased electrochemical detection signals and antibody immobilization due to their electrocatalytic effect and large specific surface area. The prepared biosensor exhibited excellent selectivity in apolipoprotein A-1 (APO-A1) detection, with an LOD of 2.2 fg mL^−1^.

#### Metal–organic frameworks (MOFs) and covalent organic frameworks (COFs)

MOFs, sometimes also called porous coordination polymers (PCPs), comprise inorganic nodes interconnected with organic ligands. With excellent physicochemical properties, large specific surface areas, controllable pore sizes, and structural/functional diversity^48^, MOFs have significant advantages as sensitive materials for biosensors. Biswas et al.^49^ synthesized Zr-trimesic acid MOF (MOF-808) in situ on carbon nanotubes (CNTs). Then, they constructed an electrochemical protein sensor by immobilizing MOF-808/CNTs (Fig. 2f) on the surface of a glassy carbon electrode (GCE) as a sensing unit for the detection of carbohydrate antigen 125 (CA-125). MOF-808 (Fig. 2e) had high electrocatalytic capability, a high specific surface area, and high protein compatibility. The linear response ranges of the fabricated biosensor for CA-125 were 0.001–0.1 and 0.1–30 ng mL^−1^, respectively. Mehmandoust et al.^50^ designed a biosensor for the rapid detection of glial fibrillary acidic protein (GFAP) based on a zeolitic imidazolate framework (ZIF-8), Au NPs, and rGO. The Au@ZIF-8@rGO nanomaterials provided high electrical conductivity and high thermal and structural stability. The biosensor specifically recognized GFAP using GFAP antibodies immobilized on the surface of the nanomaterial, achieving a very low LOD of 50 fg mL^−1^. COFs are a class of porous crystalline materials composed of organic molecules connected by covalent bonds. With a rigid skeleton, high specific surface area, and tunable pore size^51^, COFs have promising development prospects in the field of electrochemical sensors. Song et al.^52^ prepared a COF-based sandwich-type electrochemical biosensor for the efficient measurement of neuron-specific enolase (NSE). COF_Dva-TAB_ was synthesized using benzol-1,2,4,5-tetramintetrahydrochlorid (TAB) and 1,4-benzenedicarboxalaldehyde (Dva). COF_p-Fepor NH2-BPA_ was prepared using [3,3′-bipyridine]−6,6′-dicarbaldehyde (BPA), 5,10,15,20-tetrakis (4-aminophenyl)-21H, 23H-porphine (p-por-NH_2_), and FeCl_2_·4H_2_O. Then, the COF_Dva-TAB_ and Au NP/COF_p-Fepor NH2-BPA_ nanomaterials were utilized to immobilize Ab_1_ and Ab_2_, respectively. The detection signals were produced by the electrocatalytic reduction of iron porphyrin in the COF. The biosensor had an LOD of 166.7 fg mL^−1^ and a detection range of 500 fg mL^−1^–100 ng mL^−1^ for NSE.

#### Nanoenzymes

Enzymes are highly efficient catalysts with high catalytic activity and good specificity. However, natural enzymes have unavoidable limitations, such as high cost, poor stability, and strict storage conditions. Compared with natural enzymes, nanoenzymes have the advantages of low cost, stability, and mass production^53^ and have become the research focus for the next generation of enzyme mimics. Nanoenzymes can be defined as nanomaterials with enzymatic properties^53^, combining the excellent properties of nanomaterials with biocatalytic functions, and have received increasing attention in the field of biosensors. Shu et al.^32^ used Ni-Co MOF nanosheets as a nanoenzyme and modified the surface of graphene nanosheets/polyethylene terephthalate electrodes with this nanoenzyme and CNTs for the detection of AFP. The Ni-Co MOF nanosheets had a high specific surface area and could effectively bind the biotin antibody of AFP. Moreover, the nanoenzyme had high peroxidase-like activity and could effectively amplify the signal. After the formation of the antibody-AFP immunoconjugate, the catalytic ability of the MOF was greatly repressed, and the current signal was reduced, enabling sensitive detection of AFP with an LOD of 0.3 ng mL^−1^. Shu et al.^54^ prepared an electrochemical immunosensor for the highly sensitive detection of carcinoembryonic antigen (CEA) using ammoniated Cu-Ni MOF-modified electrodes. The Cu-Ni MOF had a large specific surface area and effectively immobilized CEA antibodies. In addition, the current signals of the biosensor were significantly amplified by the excellent catalytic activity of the Cu-Ni MOF. The LOD of the immunosensor was 0.16 pg mL^−1^, and the immunosensor also showed satisfactory performance in the detection of clinical serum samples. Liu et al.^55^ fabricated a label-free electrochemical immunosensor using PtPd nanocubes@MoS_2_ for hepatitis B surface antigen detection. The PtPd nanocube@MoS_2_ nanoenzymes displayed excellent peroxidase-like activity, resulting in the amplification of electrochemical signals. Due to the excellent catalytic ability and conductivity of the nanoenzymes, the biosensor exhibited high sensitivity to the hepatitis B surface antigen, including a low LOD (10.2 fg mL^−1^) and wide linear response range (32 fg mL^−1^−100 ng mL^−1^).

## Application of electrochemical protein sensors in the detection of disease markers

### Cancer marker detection

Cancer is one of the major causes of human mortality worldwide. Due to the prevalence, high recurrence rate, and potentially fatal nature of cancer, early detection and monitoring of cancer markers is very important for improving oncology patient outcomes^8^. Cancer biomarkers such as CEA^56^, AFP^45^, carbohydrate antigen 125 (CA125)^40^, prostate-specific antigen (PSA)^57^, carbohydrate antigen 72-4 (CA72-4)^58^, the extracellular domain of human epidermal growth factor receptor 2 (HER2-ECD)^59^, cytokeratin fragment antigen 21-1 (CYFRA21-1)^60^ and other proteins have been used for the detection, early screening, or prognosis assessment of many cancers, such as colorectal cancer, hepatocellular cancer (HCC), ovarian cancer, prostate cancer (PCa), gastric cancer, breast cancer, and non-small-cell lung cancer. Several electrochemical biosensors for cancer biomarker detection are described below.

**Fig. 3:**
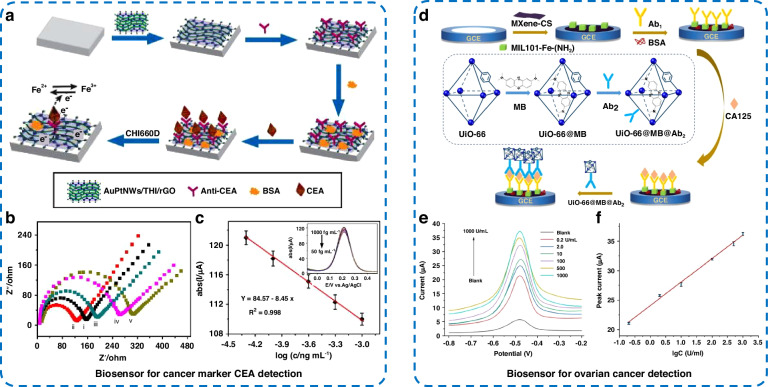
Electrochemical protein biosensors for the detection of the cancer biomarkers CEA and CA125. **a** Schematic of the construction process of the immunosensor for CEA detection. **b** Nyquist plots of the EIS. i: bare GCE; ii: AuPt NWs/THI/rGO/GCE; iii: anti-CEA/AuPt NWs/THI/rGO/GCE; iv: BSA/anti-CEA/AuPt NWs/THI/rGO/GCE; and v: CEA/BSA/anti-CEA/AuPt NWs/THI/rGO/GCE. **c** The linearity of the peak DPV currents versus the logarithm of the CEA concentration (50 fg mL^−1^–1 pg mL^−1^). The inset shows the DPV curve recorded during the electrochemical immunoassay^61^. **d** Preparation and detection process of the sandwich-type electrochemical immunosensor for CA125 detection. **e** Currents and **f** calibration curve of the sensor binding different concentrations of CA125 antigen^66^

CEA is an acidic glycoprotein produced in colorectal cancer tissues and has a molecular weight of approximately 200 kDa. CEA is a broad-spectrum tumor marker that may be an early indication of diseases such as colorectal cancer and lung cancer^56^. Therefore, the sensitive and rapid detection of CEA is important for monitoring cancer incidence and determining diagnostic efficacy. As shown in Fig. 3a, Qian et al.^61^ prepared a label-free electrochemical immunosensor using Au-Pt nanowire-functionalized thionine/reduced graphene oxide (AuPt NWs/THI/rGO) composites for the detection of CEA. The AuPt NW/THI/rGO nanomaterials promoted both antibody immobilization and charge transport (Fig. 3b). The prepared biosensor exhibited excellent properties for CEA detection, including a low LOD of 6 fg mL^−1^ at a signal-to-noise ratio of 3 (S/N = 3) and satisfactory linearity (Fig. 3c), reproducibility, stability, and selectivity. Importantly, the prepared immunosensor was compared with the ELISA technique for the identification of CEA in three human diluted blood samples, and the experimental results showed that the relative errors of both approaches ranged from −3.74% to 3.59%, indicating that the immunosensor could be used for the clinical detection of CEA. Wang et al.^62^ fabricated an electrochemical protein biosensor for CEA detection by depositing Au nanoparticles and β-cyclodextrin on polyaniline-modified MXene (Au-β-CD/MXene@PANI) to construct a high-charge-transfer interface and introduce a suitable biocompatible carrier to immobilize the antibody or antigen. Due to the fast electron mobility of the MXene@PANI composites and the outstanding antibody immobilization ability of Au-β-CD, the practical detection range of this sensor for CEA was 0.5–350 ng mL^−1^, with an LOD of 0.0429 ng mL^−1^. The properties of the biosensor for the detection of CEA in real human serum were investigated using the standard addition approach, and the calculated recoveries of the sensor in clinical serum samples ranged from 97.52% to 103.98%, indicating high detection accuracy and potential for large-scale application.

AFP, a glycoprotein with a molecular weight of approximately 70 kDa, is one of the most reliable tumor biomarkers for the diagnosis of HCC^63^. The serum concentrations of AFP in healthy adults are normally less than 25 ng mL^−1^, but the level is clearly elevated in patients with hepatocellular carcinoma^64^. Early detection and accurate diagnosis of AFP are facilitate effective treatment for hepatocellular carcinoma. Li et al.^45^ prepared a sandwich-type electrochemical protein biosensor for AFP detection using amino group-containing graphene modified with Au nanoparticles (Au NPs/NH_2_-GS) as a conductive matrix material and Au@Pt dendritic nanorods modified with amino group-containing MoSe_2_ nanosheets (Au@Pt DNRs/NH_2_-MoSe_2_ NSs) as a secondary antibody (Ab_2_) label with good catalytic properties. The current response of the immunosensor exhibited good linearity with respect to AFP in the range of 10–200 ng mL^−1^. The LOD of the biosensor was 3.3 fg mL^−1^ (S/N = 3), with good reproducibility, stability, and selectivity. AFP was detected in real human serum specimens using the standard addition approach, and the spiked recoveries were 99.91–100.8%, with a relative standard deviation of <2.36%. In addition, the prepared sandwich-type biosensor was used to test the same AFP samples with the ELISA technique, and the relative deviation of both methods was <1%, which indicated that the prepared immunosensor has potential for clinical application. Luo et al.^65^ constructed an AFP immunosensor using Au NPs and PANI nanowires functionalized with polyethylene glycol (PEG). This sensor combined the inherent electroactivity and conductivity of PANI, the high conductivity and biocompatibility of Au NPs, and the excellent antifouling ability of PEG. The biosensors utilized the redox currents of PANI because these response signals did not require additional signal reagents, and antibiofouling properties were observed in complex serum samples. The sensor had an ultralow LOD (0.007 pg mL^−1^), and its usefulness was verified by the detection of AFP in real serum specimens.

CA125, a macromolecular glycoprotein, is the most commonly used clinical biomarker in screening for ovarian cancer^40^. In addition, elevated concentrations of CA125 in the blood have been associated with malignant diseases such as breast, gastric, lung, and intestinal cancers^66,67^. The concentration of CA125 in the blood of healthy adults is less than 35 U mL^-1^^67^. Pan et al.^66^ developed a dual MOF sandwich-type MXene/MIL-101(Fe)-NH_2_/UiO66@methylene blue (MB) electrochemical immunosensor (Fig. 3d) for the assessment of CA125 in serum. MXene increased the conductivity of the electrode. MIL-101(Fe)-NH_2_, which is rich in amino groups, increased the loading of Ab_1_, while the catalytically active Fe metal sites could catalyze redox reactions. UiO66@MB was used for signal amplification, and the loading ratio of Ab_2_ was increased at the same time. The dual MOF sandwich immunosensor showed marked sensitivity and good selectivity for the determination of CA125, with a wide detection range (Fig. 3e, f) and an LOD of 0.006 U mL^−1^. The prepared biosensors were in close agreement with the results of the chemiluminescence immunoassay commonly used in hospitals. Dong et al.^68^ designed and synthesized MOF, COF, and CNT hybrid materials for the construction of label-free CA125 immunosensors. TPN-COF/CNT was prepared using a mixture of terephthalonitrile (TPN), ZnCl_2_, and CNTs by a milling and calcination process. Then, it was placed in a synthetic system containing Ce(NO_3_)_3_·6H_2_O and trimeric acid to obtain Ce-MOF/TPN-COF/CNT. The hydrogen bonding and noncovalent bonding interactions between the triazine ring and the trimeric acid facilitated the immobilization of immune complexes on the Ce-MOF/TPN-COF/CNT-modified carbon paste electrodes (CPEs), amplifying the current responses. The linear response range of the prepared immunosensor was 0.0001–100 U mL^−1^, and the LOD was as low as 0.000088 U mL^−1^.

**Fig. 4:**
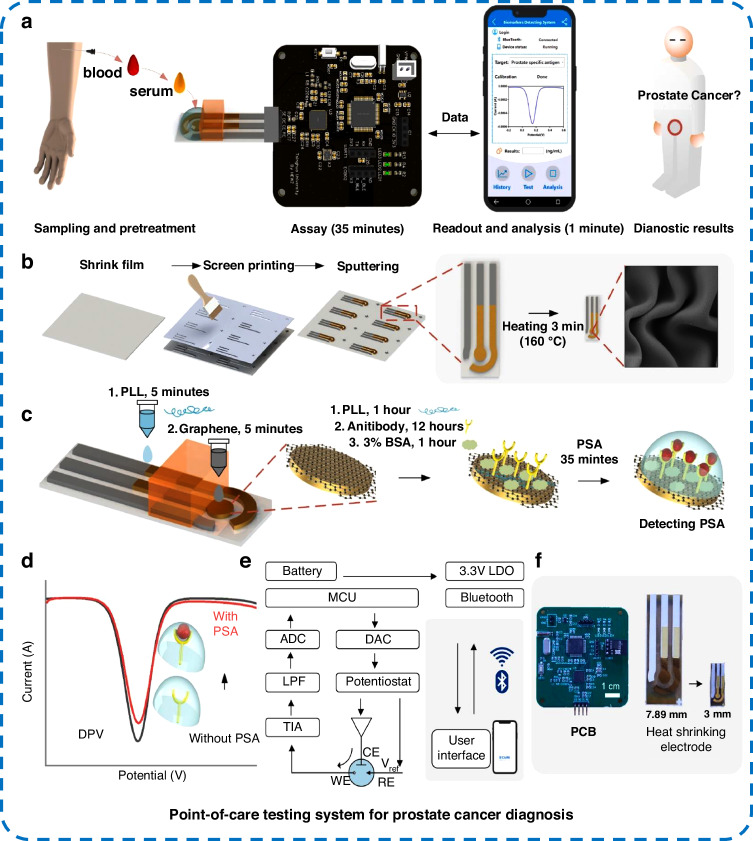
Shrinking polymer-based electrochemical protein biosensor system for detection of the PCa biomarker PSA. **a** PSA detection process, **b** shrinking polymer-based three-electrode system, **c** sensor surface modification process, **d** detection of PSA by decreasing the peak current, **e** block diagram of the point of care (POC) system, and **f** photographs of printed circuit boards (left) and electrodes before (center) and after (right) shrinking^70^

PCa is a malignant tumor that poses a serious threat to men’s health. In 2020, the International Agency for Research on Cancer (IARC) announced that PCa ranked second in male cancer incidence and fifth in mortality^57^. PSA is the most effective biomarker for detecting prostate cancer^57,69^. The serum PSA concentration in healthy men is generally less than 4 ng mL^−1^, and a serum PSA concentration greater than 4 ng mL^−1^ is associated with a risk of cancer^57,70^. Therefore, PSA detection at the ng mL^−1^ level is necessary. As shown in Fig. 4, Ye et al.^70^ reported a shrink polymer-based immunosensor for the first time and integrated it into a compact portable system for PSA detection. Immunosensors were made by sputtering Au film onto a shrinking polymer, which was then heated and shrunk into a small-sized electrode with wrinkles (Fig. 4b). The wrinkles on the electrode surface could be adjusted by varying the thickness of the sputtered Au film, which had a large specific surface area and promoted antigen–antibody binding. The sensitivity of the biosensor was improved by air plasma treatment and modification with self-assembled graphene. The LOD for PSA in serum was 0.38 fg mL^−1^, with a linear response range of 10 fg mL^−1^–1000 ng mL^−1^. The results of the biosensor were similar to those of a commercial chemiluminescence instrument (Autolumo A2000 plus), confirming the feasibility of clinical application. The biosensor was integrated into a portable testing system that could be controlled through software (Fig. 4a, e) and was suitable for real-time diagnostics. Xia et al.^71^ developed a dual-signal sandwich-type electrochemical protein biosensor using CoTe_2_@CeO_2_ nanomaterials as electrocatalytic labels. The effective interaction between CoTe_2_ and CeO_2_ increased the ability of the nanocomposites to catalyze the oxygen evolution reaction (OER), and Te^-^ oxidation was obvious in the OER process, which produced large OER signals and distinct oxidation peak signals. Through the CoTe_2_ oxidation peak currents (I_peak_) and OER currents (I_OER_) during the electrocatalytic OER process, the sandwich-type biosensor could be used for dual-signal detection of PSA with a wide linear detection range (0.002–200 ng mL^−1^) and low LOD (I_peak_: 8.26 fg mL^−1^, I_OER_: 448 fg mL^−1^). In addition to enabling sensitive PSA detection, this method enables validation between the two detection signals, increasing the accuracy. The performance parameters of electrochemical protein biosensors related to the detection of colorectal, hepatocellular, ovarian, and prostate cancers are summarized in Table 1.

**Table 1:** Performance of electrochemical protein sensors for cancer detection

Cancer	Biomarker	Biosensor	Materials	LOD	Range	Refs.
Colorectal cancer, lung cancer, and so on	CEA	Glassy carbon electrode	AuPt NWs/THI/rGO	6 fg mL^−1^	50 fg mL^−1^–100 ng mL^−1^	^61^
	CEA	FTO	Au-β-CD/MXene@PANI	0.0429 ng mL^−1^	0.5–350 ng mL^−1^	^62^
	CEA	Carbon printed electrode	Cu-Ni MOF	0.16 pg mL^−1^	0.5 pg mL^−1^–500 ng mL^−1^	^54^
	CEA	Glassy carbon electrode	ZrO_2_-rGO-IL	1.23 fg mL^−1^	25 fg mL^−1^–25 ng mL^−1^	^112^
Hepatocellular cancer	AFP	Glassy carbon electrode	Au NPs/NH_2_-GS, Au@Pt DNRs/NH_2_-MoSe_2_ NSs	3.3 fg mL^−1^	10 fg mL^−1^–200 ng mL^−1^	^45^
	AFP	Glassy carbon electrode	PEG/Au NPs/PANI	0.007 pg mL^−1^	0.01 pg mL^−1^–1 ng mL^−1^	^65^
	AFP	Glassy carbon electrode	Fe_3_O_4_/MWCNTs-COOH/Au NPs	1.09034 pg mL^−1^	1 pg mL^−1^–10 μg mL^−1^	^113^
	AFP	Gold electrode	Chitosan@Ag/CeO_2_	0.57 pg mL^−1^	10^−12^–10^−6 ^g mL^−1^	^114^
Ovarian cancer	CA125	Glassy carbon electrode	MXene/MIL-101(Fe)-NH_2_	0.006 U mL^−1^	0.2−1000 U mL^−1^	^66^
	CA125	Carbon paste electrode	Ce-MOF/TPN-COF/CNT, UiO66@MB	0.000088 U mL^−1^	0.0001-100 U mL^−1^	^68^
	CA125	Glassy carbon electrode	CMK-3(Au/Fc@MgAl-LDH)n multilaminate nanocomposites	0.004 U mL^−1^	0.01–1000 U mL^−1^	^115^
	CA125	Glassy carbon electrode	3DrGO-MWCNTs/polyamidoamine/Au NPs, Suc-CS@MNPs-TB	6 μU mL^−1^	0.0005–75 U mL^−1^	^116^
Prostate cancer	PSA	Shrink polymer electrode	Self-assembled graphene	0.38 fg mL^−1^	10 fg mL^−1^–1000 ng mL^−1^	^70^
	PSA	Glassy carbon electrode	Au NPs, CoTe_2_@CeO_2_	I_peak_: 8.26 fg mL^−1^ I_OER_: 448 fg mL^−1^	0.002–200 ng mL^−1^	^71^
	PSA	Glassy carbon electrode	Au NPs, (NiFeCo)_2_P@Au/CeO_2_	I_HER_: 116.86 fg mL^−1^ I_OER_: 39.05 fg mL^−1^	0.0001–100 ng mL^−1^	^57^
	PSA	Glassy carbon electrode	AgPt@Pt HNs/PPy NS	120.3 fg mL^−1^	0.0005–50 ng mL^−1^	^117^

### Detection of viral infectious disease markers

Infectious diseases, which are caused by pathogens such as bacteria, viruses, parasites, or fungi, can be transmitted from person to person, from animal to animal, or from animal to person. Over the past decade, viral infectious diseases (such as coronavirus disease, dengue fever, Ebola virus disease, and Zika virus disease^5,72^) have been ravaging the globe, presenting a severe hazard to world public health and economic growth. The detection of infectious disease at an early stage allows timely treatment and effectively reduces the mortality rate. Therefore, the use of highly specific and sensitive biomarkers to detect sources of infection is essential for the prevention of infectious disease epidemics and the diagnosis of infectious diseases. Severe acute respiratory syndrome coronavirus 2 (SARS-CoV-2) antibodies and antigens^73^, anti-dengue antibodies^74^, and inactivated viruses^75^ can be used as biomarkers of the corresponding infectious diseases to detect the source of infection.

**Fig. 5:**
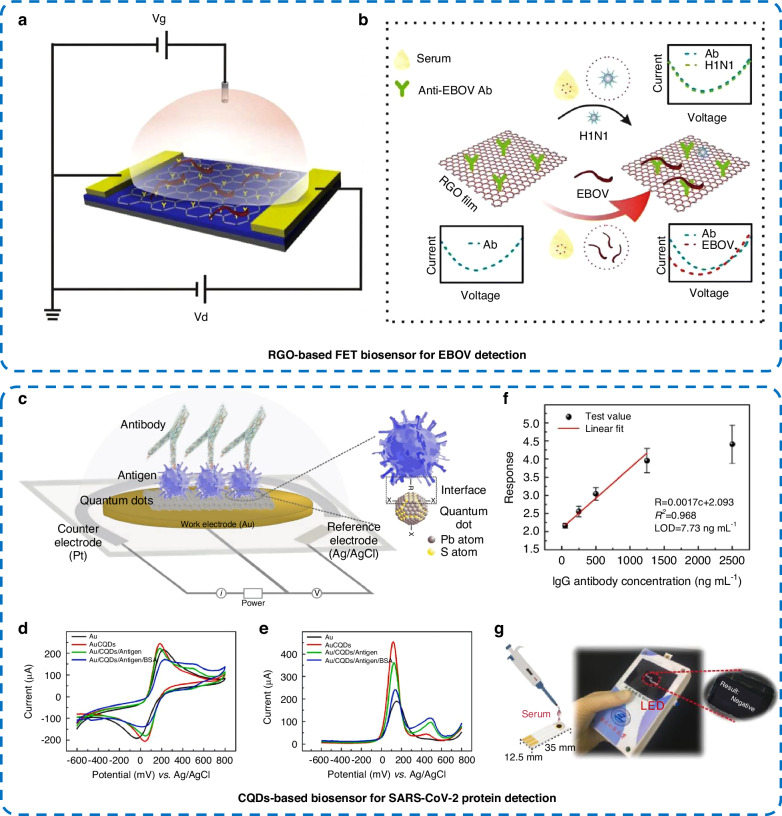
Electrochemical protein biosensors for the diagnosis of EBOV and SARS-CoV-2 viral infections. **a**, **b** Schematic diagram of an RGO-based FET for EBOV detection^75^. **c** Schematic diagram of the CQD-modified electrode used as a biosensor for SARS-CoV-2 protein detection. **d** CV and **e** DPV characterization of bare Au electrodes, Au/CQDs, Au/CQDs/antigen, and Au/CQDs/antigen/BSA in the presence of 1× PBS containing 5 mM [Fe(CN)_6_]^3−/4−^ and 0.5 M KCl as redox medium. **f** Linear fit of the recombinant antibody concentration to the response; and **g** handheld test system prototype^12^

Dengue is a major public health challenge in tropical and subtropical countries. Dengue virus is carried by *Aedes aegypti* mosquitoes and spreads rapidly, affecting 400 million people worldwide each year^76^. Therefore, the early and rapid diagnosis of dengue fever is needed to prevent large-scale epidemics. Prakash et al.^77^ prepared a high-performance immunosensor based on Au nanorod-modified graphitic carbon nitride (Au NRs-g-C_3_N_4_) for the screening of the dengue biomarker nonstructural protein 1 (NS1-Dengue). NS1-Dengue antibodies were immobilized on the surface of the Au NR-g-C_3_N_4_-modified GCE and used as an impedance sensing probe to detect NS1 antigens. The prepared immunosensor showed a good electrocatalytic response in quantifying NS1 antigens in PBS buffer solution and human real serum samples. With a broad linear response range (0.6–216 ng mL^−1^) and an LOD of 0.09 ng mL^−1^, the sensor was expected to be used as a potential barrier detection technique for the diagnosis of dengue virus in biological samples. Zika virus (ZIKV) is a virus with a high transmission rate in the tropical regions of Asia, the Americas, and Africa. In addition to being transmitted by *A. aegypti* mosquitoes, ZIKV can be transmitted through saliva, urine, and other routes, which makes its prevention and control more difficult^78^. Therefore, there is an urgent need for ZIKV detection techniques. Faria et al.^79^ prepared an electrochemical protein biosensor for the testing of anti-ZIKV antibodies by using nonstructural protein 1 (NS1-ZIKV)-derived recombinant protein-functionalized magnetic beads and horseradish peroxidase (HRP)-labeled anti-IgG antibodies. The detection range of this biosensor for anti-ZIKV antibodies was 0.01–9.80 × 10^5 ^pg mL^−1^ (r^2^ = 0.982), and the LOD was 0.48 pg mL^−1^. In addition, the prepared immunosensor was effective in the screening of anti-ZIKV antibodies in real serum samples. Ebola Virus Disease (EVD) is a deadly epidemic with a lethality rate of up to 50%^80^. EVD is a zoonotic disease caused by the Ebola virus (EBOV). In addition to being transmitted by fruit bats and nonhuman primates, EBOV can be transmitted through direct contact with infected bodily fluids^80^. The detection of carriers of EBOV is necessary for effective disease management. Zhang et al.^75^ proposed an immunoassay for the direct detection of inactivated EBOV using RGO-modified FET. The FET surface was modified with RGO, and an antibody against the EBOV glycoprotein was immobilized on the surface of the FET chip, enabling the direct detection of EBOV (Fig. 5a, b). EBOV has a high binding affinity for antibodies, and the conductance of the FET chip changed significantly upon EBOV binding to the antibody, whereas other viruses without specific binding cause negligible changes in conductance. The FET chip could measure EBOV in a concentration range of 2.4 × 10^−12^–1.2 × 10^−7^ g mL^−1^ with an LOD as low as 2.4 pg mL^−1^.

Coronavirus disease 2019 (COVID-19) is an infectious disease caused by severe acute respiratory syndrome coronavirus 2 (SARS-CoV-2) infection^81^. With a significant increase in the number of cases around the world since its first appearance in 2019, the rapid spread of COVID-19 has had a major influence on human health, the economy, education, and social development worldwide^82^. COVID-19 can be transmitted from person to person through contact, droplet, and airborne routes. The major symptoms of COVID-19 infection are cough, fever, and shortness of breath^83^. Therefore, there is an unprecedented demand for the design of devices capable of the rapid and highly selective diagnosis of COVID-19. As shown in Fig. 5c, Liu et al.^12^ designed an all-solid-state SARS-CoV-2 electrochemical protein biosensor with PbS colloidal quantum dot (CQD)-modified electrodes. Taking advantage of the surface organic ligand interchangeability of PbS CQDs, an electronic label of SARS-CoV-2 protein was constructed on a gold working electrode. Under an applied alternating voltage, the PbS CQDs exhibited unique charging and discharging effects due to the quantum confinement regime. The specific binding of the SARS-CoV-2 antigen to the antibody was converted to distinct electrical signals (Fig. 5d, e). The protein biosensor had a linear response range of 50–1250 ng mL^−1^ (Fig. 5f) and a lower LOD of 7.73 ng mL^−1^. In addition, it discriminated between normal samples and patients with an accuracy of approximately 90%. A prototype handheld test system was prepared by integrating the sensor with the circuit system (Fig. 5g). The test system made it possible to read the results in less than 30 s, which was approximately 10 times faster than the detection speed of commercial CGIA kits. In addition, Wei et al.^84^ developed a graphene FET-based biosensor chip for SARS-CoV-2 antibody detection. The detection chip was fabricated by a semiconductor fabrication process. The SARS-CoV-2 spike S1 protein was immobilized on a graphite surface as a probe for detecting SARS-CoV-2 antibodies. The immunocomplex of the SARS-CoV-2 spike S1 protein with the antibody was translated into electrical signal changes in the graphene channel. The LOD of this FET biosensor for SARS-CoV-2 antibodies was 390 ag mL^-1^. Due to the heavy impact of the worldwide COVID-19 pandemic on countries since 2019, many studies have been dedicated to the design and development of electrochemical protein sensors for the rapid and specific detection of SARS-CoV-2. We summarize the performance parameters of electrochemical biosensors related to SARS-CoV-2 antibody/antigen detection in Table 2.

**Table 2:** Performance of electrochemical protein sensors for detecting SARS-CoV-2

Biosensors	Materials	Biomarker	LOD	Response time	Range	Refs.
All-solid-state protein biosensor	CQDs	SARS-CoV-2 antibody	7.73 ng mL^−1^	30 s	50–1250 ng mL^−1^	^12^
Cotton-tipped electrochemical immunosensor	Carbon nanofiber	SARS-CoV-2 antigen	0.8 pg mL^−1^	–	0.1–10^6 ^pg mL^−1^	^118^
Paper-based electrochemical biosensor	Graphene oxide	SARS-CoV-2 antibody	1 ng mL^−1^	30 min	1–1000 ng mL^−1^	^73^
Screen-printed carbon electrodes	GQDs@AuPt	SARS-CoV-2 antigen	60.14 ng mL^−1^	–	0.05–1.5 μg mL^−1^	^119^
FET	Graphene	SARS-CoV-2 antibody	2.6 aM	<2 min	5 aM–5 pM	^84^
Electrochemical biosensor chip	MOF nanohybrids	SARS-CoV-2 antigen	6.68 fg mL^−1^ (in buffer), 6.20 fg mL^−1^ (in serum)	<5 min	10–10^7^ fg mL^−1^	^120^
FET	Single-walled CNT	SARS-CoV-2 antigen	0.55 fg mL^-1^ (S antigen), 0.016 fg mL^−1^ (N antigen)	<5 min	0.55 fg mL^−1^–55 μg mL^−1^ (S antigen), 0.016 fg mL^−1^–16 μg mL^−1^ (N antigen)	^121^
3D electrodes	rGO	SARS-CoV-2 antibody	2.8 × 10^−15 ^M (spike S1), 16.9 × 10^−15 ^M (receptor-binding-domain)	<15 s	1 × 10^−12^–10^−8^ M (spike S1), 1× 10^−12^–2 × 10^−8 ^M (receptor-binding-domain)	^122^
Pencil graphite electrodes biosensor	Au NPs-cysteamine	SARS-CoV-2 antigen	229 fg mL^−1^	6.5 min	10^−12^–10^−10 ^g mL^−1^	^123^
FET	MoS_2_	SARS-CoV-2 antibody	–	–	10^−9^–10^−3 ^μg μL^−1^	^124^
Organic electrochemical transistors	Poly(3,4-ethylenedioxythiophene)–polystyrene sulfonate	SARS-CoV-2 antigen	–	<10 min	1 fg mL^−1^–1 µg mL^−1^	^125^
Duplex electrochemical microfluidic sensor	Carbon black and gold	SARS-CoV-2 antibody	28 ng mL^−1^ (N-Ig G), 15 ng mL^−1^ (S-Ig G)	6.5 min	30–750 ng mL^−1^ (N-Ig G), 20–1000 ng mL^−1^ (S-Ig G)	^126^

### Inflammatory marker detection

Inflammation is the body’s defense response to stimuli and manifests as redness, swelling, heat, pain, and dysfunction. Controlled inflammation is a necessary process for wound healing, tissue repair, and defense against invasion by foreign pathogens^85^. In most cases, inflammation subsides spontaneously without residual tissue damage or fibrosis, but inflammation can also, in many individuals, lead to long-term progressive disability and ultimately premature death^86^. Therefore, early diagnosis and effective treatment of inflammation are needed to prevent long-term negative effects on the work and quality of life of patients. Proteins such as interferon-γ (IFN-γ)^87^, tumor necrosis factor-alpha (TNF-α)^88^, anti-cyclic citrullinated peptide antibody (anti-CCP)^89^, and eosinophilic cationic protein (ECP)^90^ are biomarkers of inflammatory diseases such as autoimmune hepatitis, rheumatoid arthritis (RA), and allergic rhinitis (AR) and are used for diagnosis.

**Fig. 6:**
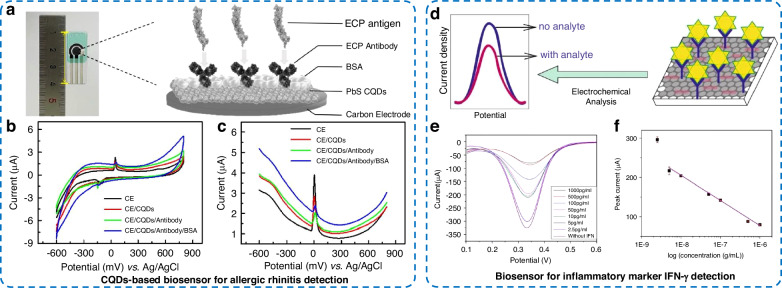
Electrochemical protein biosensors for the detection of the inflammatory biomarkers ECP and IFN-γ. **a** Bare carbon electrode (CE)/CQDs/antibody/BSA electrode image and schematic diagram of the CQD-modified electrode. **b** CV and **c** DPV characterization of CE, CE/CQDs, CE/CQDs/antibody and CE/CQDs/antibody/BSA in the presence of 1×PBS^93^. **d** Schematic of the immunosensor, where IFN-γ binding reduces the measurement current. **e** DPV characterization of the immunosensor at concentrations ranging from 0 to 1000 pg mL^−1^. **f** The corresponding linear fit^99^

AR is the most common allergic respiratory disease and is often involves symptoms such as sneezing, nasal congestion, itching, and a runny nose^91,92^. Severe AR seriously affects people’s work and quality of life. Liu et al.^93^ selected semiconductor CQD-modified carbon electrodes to construct an electrochemical protein biosensor for the detection of ECP, a specific biomarker for AR (Fig. 6a). CQDs exert significant size and quantum effects that promote the immobilization of biomolecules on the electrode surface and convert the specific binding of ECP antigens to antibodies into electrical signals (Fig. 6b, c). With an LOD of 0.508 pg mL^−1^ for the ECP antigen and a detection time of approximately 30 s, this electrochemical biosensor has the potential to enable rapid, noninvasive, in vitro diagnosis of AR. RA is a chronic inflammatory joint disease that can lead to the destruction of cartilage and synovial joints and can occur at any age^89,94^. Early diagnosis and treatment of RA can greatly slow joint damage in patients, thus preventing irreversible disability. Fan et al.^89^ developed a peptide electrochemical sensor for the detection of anti-CCP antibodies for the diagnosis of RA. A new citrulline-modified peptide was first prepared and attached to the surface of the Au working electrode of the sensing chip by a self-assembled monolayer. The impedance at different frequencies was measured to characterize the electrical performance of the prepared electrochemical biosensor. The excellent sensitivity of the prepared peptide-based electrochemical sensor was verified by comparison with a commercial anti-CCP ELISA kit for the screening of serum samples from clinical patients. Hashimoto thyroiditis (HT) is an autoimmune thyroid disease also called chronic lymphocytic thyroiditis. It can be diagnosed in patients of any age, and its incidence increases with age^95^. The diagnosis of HT is usually made by clinical features as well as positive serum antibodies to thyroid antigens (including thyroid peroxidase)^95^. Beitollahi et al.^96^ reported for the first time an electrochemical biosensor capable of effectively detecting anti-thyroid peroxidase antibodies (anti-TPO) for the rapid and routine analysis of HT. A sandwich-type electrochemical protein biosensor was manufactured by first depositing Au NPs on the surface of CPEs and then sequentially immobilizing human recombinant TPO (Ab_1_), anti-TPO, and HRP-anchored anti-TPO secondary antibody (HRP-Ab_2_) on the surface. The catalytic properties of HRP significantly increased the reduction detection signal of the sensor. The conditions of the sensor were optimized to provide a wide linear response range (0.02–60.0 μg mL^−1^), a low LOD (6 ng mL^−1^), and satisfactory reproducibility and stability for anti-TPO detection.

Inflammatory cytokines such as TNF-α, IFN-γ and oncostatin M (OSM) have been found to be associated with many infectious and inflammatory diseases (such as RA, autoimmune hepatitis, and inflammatory bowel disease)^87,97,98^ and can be used as markers for inflammatory disease detection. Pinto et al.^88^ developed a label-free ultrasensitive immunosensor for the detection of TNF-α. Au electrodes functionalized with self-assembled monolayers of sulfosuccinimidyl 6-(3’-(2-pyridyldithio) propionamido) hexanoate effectively immobilize antibodies and offered improved analytical capabilities. The immunosensor was shown to be the only method that could detect TNF-α in body fluids such as cerebrospinal fluid and tears. With an LOD of 0.085 pg mL^−1^ in tears and 2 pg mL^−1^ in cerebrospinal fluid and serum, it has the potential for the early diagnosis of inflammation and monitoring of TNF-α levels during treatment. Mampallil et al.^99^ designed an immunosensor based on Au NR and rGO composite (Au NR-rGO)-modified indium tin oxide electrodes. Antibodies were anchored to the nanomaterial-modified electrodes for the detection of IFN-γ (Fig. 6d). As shown in Fig. 6e, f, the electrochemical protein biosensor had a linear dynamic range of 5–1000 pg mL^−1^ and an LOD of 2.5 pg mL^−1^ for the specific detection of IFN-γ. Andrade et al.^100^ developed a nanoengineered immunosensor. The biosensor electrode surface was modified with a polypyrrole conductive layer, Au nanoparticles, and bioreceptor molecules (anti-OSMR antibody or oncostatin M receptor (sOSMR) protein) for the detection of the sOSMR protein or anti-OSMR antibody. The anti-OSMR antibody-based sensor demonstrated sensitivity for the detection of sOSMR protein with an LOD of 0.42 pg mL^−1^ and a linear response between 0.005 and 500 pg mL^−1^.

**Fig. 7:**
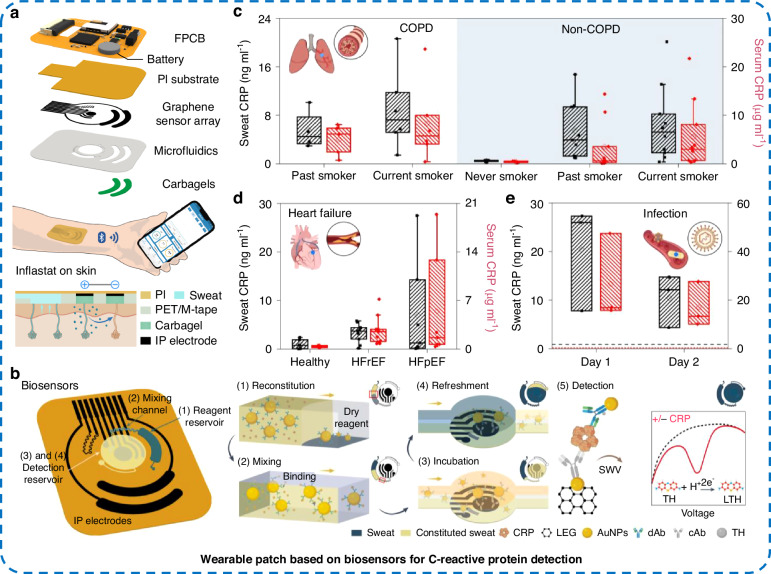
Wearable patch for monitoring protein biomarkers of inflammation. **a** Schematic of a wearable skin patch for monitoring proteins in sweat, **b** mechanisms for electrochemical detection of proteins in sweat in combination with skin interface microfluidic modules, CRP protein levels in sweat and serum samples from patients with **c** COPD, **d** HF, or **e** acute infection caused by inflammation (HFrEF: HF with reduced ejection fraction, HFpEF: HF with preserved ejection fraction)^104^

Wearable electrochemical biosensor systems enable the sensitive real-time monitoring of human physiological biomarkers and are a popular research focus in this field^101,102^. However, wearable electrochemical biosensors to date tend to be focused on monitoring small molecules, such as glucose and lactate^103^. Integrating electrochemical protein biosensors into wearable devices will be of interest to researchers^104^. C-reactive protein (CRP) is a protein biomarker of inflammation. The concentration of CRP increases dramatically when the body is exposed to infection or tissue damage, which provides a way to assess patients’ inflammatory status. Gao et al.^104^ reported a wearable patch for monitoring CRP in sweat (Fig. 7). As shown in Fig. 7a, the wearable skin patch mainly consisted of an iontophoretic module, a microfluidic module, and a sensor array for monitoring CRP, temperature, and other information. CRP was detected through a sandwich-type electrochemical biosensor in the sensor array (Fig. 7b). Capture antibodies were immobilized on the surface of the Au NP-modified graphene working electrode to capture CRP, and detection antibodies were immobilized on the Au NPs to amplify electrochemical signals. The wearable patch was used to monitor systemic inflammation in chronic obstructive pulmonary disease (COPD), HF, or acute infectious states (Fig. 7c–e). The CRP concentration in sweat obtained by the patch was strongly correlated with that in serum, indicating that the detection of CRP in sweat using wearable patches has broad application prospects in the noninvasive monitoring of systemic inflammation.

### Detection of other disease markers

**Fig. 8:**
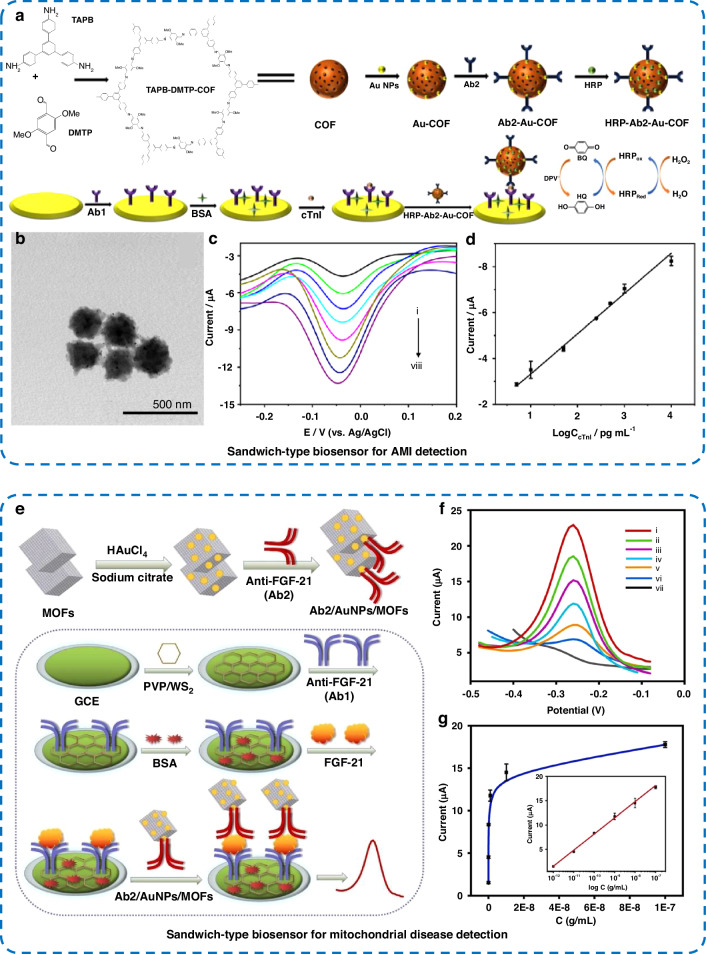
Electrochemical protein biosensors for the detection of the AMI biomarker CTnI and the mitochondrial disease biomarker FGF-21. **a** Schematic of the synthesis of HRP-Ab_2_-Au-COF and preparation of the cTnI detection biosensor, **b** TEM image of HRP-Ab_2_-Au-COF, **c** DPV response curves of the biosensor (i-viii: the concentrations of cTnI range from 0 to 10 ng mL^−1^) and **d** linear relationship between currents and the logarithm of cTnI concentrations^106^. **e** Schematic of FGF-21 detection based on PVP/WS_2_ nanosheet-modified electrodes and Au NP/MOFs. **f** DPV curves of the sensor for FGF-21 detection (vi-i: 1 pg mL^−1^–100 ng mL^−1^, vii: blank) and **g** the corresponding calibration curve^108^

Acute myocardial infarction (AMI) is the most severe type of coronary heart disease and poses a serious threat to human health. After myocardial injury in AMI patients, cardiac troponin I (CTnI) levels increase and persist, providing a window for AMI screening^105^. CTnI is considered the gold standard for AMI diagnosis. Huang et al.^106^ prepared a COF-based probe and used it for the immunoassay of CTnI (Fig. 8a). The high porosity of the COF allowed it to encapsulate a large amount of HRP, which could markedly amplify the electrochemical reduction signals. Moreover, the COF had excellent biocompatibility and could effectively maintain enzyme activity and stability. The signal probe HPR-Ab_2_-Au-COF (Fig. 8b) integrates target recognition and signal amplification. The peak currents of the sandwich biosensor were linearly related to the logarithm of 5–10 ng mL^−1^ CTnI (Fig. 8c, d), with an LOD of 1.7 pg mL^−1^. Alzheimer disease (AD) is a persistent neurological dysfunction and one of the most common causes of dementia worldwide, causing great suffering to patients and society^107^. Escosura-Muñiz et al.^107^ reported the use of conformationally altered p53 peptides as biomarkers for AD and the application of bifunctional core-cell Au@Pt/Au NPs as a new label for p53 peptides. An anti-p53 monoclonal antibody was coupled to the surface of the Au@Pt/Au NPs, and the p53 peptide was immobilized on magnetic beads. Electrocatalysis was used to measure AD biomarkers in a competitive immunoassay using a magnetic bead platform with an LOD as low as 66 nM. Mitochondrial diseases are progressive incurable diseases and multisystem disorders with variable clinical presentations that are often difficult to diagnose^108,109^. Serum fibroblast growth factor-21 (FGF-21) is a new specific marker for mitochondrial disease and might facilitate diagnosis. Guo et al.^108^ used a polyvinylpyrrolidone-tungsten disulfide (PVP/WS_2_)-modified electrode and an electrochemical sandwich-type immunosensor with Au NP/MOF nanoenzymatic probes for the detection of FGF-21 (Fig. 8e). The immunosensor has excellent sensitivity for FGF-21 detection, with a dynamic range of 1 pg mL^−1^–100 ng mL^−1^ (Fig. 8f) and an LOD of 0.24 pg mL^−1^. The peak currents of the electrochemical biosensor exhibited a favorable linear correlation with the logarithm of the FGF-21 concentration (Fig. 8g). Acute kidney injury (AKI) is a common severe disease with a high mortality rate^110^. Lipocalin 2 (LCN 2) is a protein with a molecular weight of 25 kDa that mainly exists in the kidney, liver, epithelial cells, and other tissues. LCN 2 is considered a promising biomarker for the prediction of kidney-related diseases^111^. Kao et al.^111^ prepared NiO NPs-modified CeCuO_x_ (NiO NPs/CeCuO_x_) thin film-based biosensors. First, CeCuO_x_ and NiO were deposited successively on a silicon substrate to prepare the electrode, and then, anti-LCN 2 was immobilized on its surface to detect the presence of LCN 2. The immunosensor had a low LOD (4.23 ng mL^−1^), a linear response range of 25–400 ng mL^−1^, and high selectivity.

## Summary and outlook

With the unprecedented rapid growth of electrochemical protein biosensor technology, research achievements in the rapid and specific detection of biomarkers of cancer, viral infectious disease, inflammation, and other diseases continue to emerge. Researchers have optimized the molecular recognition function of electrochemical protein sensors by exploiting different sensitive nanomaterials (such as metal NPs, QDs, CNTs, graphene, MOFs, and composite nanomaterials) for chemical modification of the electrodes. Researchers have also optimized the signal transduction function according to the structure of the transducer devices (including three electrodes and FETs). The above strategies have increased the specificity and sensitivity of electrochemical protein biosensors for use in the detection of disease biomarkers.

Electrochemical protein sensors have the potential for miniaturization, high throughput, and smart use. These sensors can be integrated with microfluidic systems, lab-on-a-chip systems, and small circuit systems in the future, portable detection systems and point-of-care testing (POCT) analysis platforms are expected to be designed and implemented. Several challenges need to be addressed to increase the performance and usability of electrochemical protein sensors for disease biomarker detection. (i) In the detection process for actual human samples, body fluid samples, such as whole blood, serum, and nasopharyngeal swabs, have a complex composition, which hinders the accuracy and reliability of electrochemical protein biosensors. Single biomarker detection may also result in insufficient biosensor accuracy. The simultaneous detection of multiple biomarkers in a single sensor is expected to increase the accuracy of biosensors in diagnosing diseases. (ii) Wearable smart electrochemical protein sensors based on flexible electrodes can provide real-time and noninvasive health information and have attracted a wide range of research interests. (iii) In addition, extensive validation studies of electrochemical protein biosensors are required to support their clinical application.

In summary, electrochemical protein sensors show promise for disease detection in the future. Technical progress has mainly focused on miniaturization, long-term continuous monitoring, and increased sensitivity, specificity, and biocompatibility. This progress will contribute to the development of more efficient, accurate, and practical applications of electrochemical protein biosensors for life and health, home health care, clinical diagnosis, treatment, and prognosis assessment.
